# Revolutionary ctDNA-Guided Therapy Adaptation in *ESR1*-Mutated Advanced Breast Cancer: Insights from the Initial SERENA-6 Trial

**DOI:** 10.34133/cancomm.0012

**Published:** 2026-03-13

**Authors:** Lulu Yang, Ning Zhu, Ying Yuan, Jing Zhao

**Affiliations:** ^1^ Department of Medical Oncology, The Second Affiliated Hospital of Zhejiang University School of Medicine, Hangzhou, Zhejiang, P. R. China.; ^2^ Department of Medical Oncology, Ninghai First Hospital, Ningbo, Zhejiang, P. R. China.

Estrogen receptor 1 mutations (*ESR1*m) are acquired in approximately 30% to 40% of patients with hormone-receptor-positive (HR^+^), human epidermal growth factor receptor 2-negative (HER2^−^), advanced breast cancer (ABC) who have developed resistance to first-line aromatase inhibitor (AI) plus cyclin-dependent kinase 4/6 inhibitor (CDK4/6i) therapy [1]. Although treatment decisions were traditionally guided by radiological progression, circulating tumor DNA (ctDNA) monitoring can detect emergent *ESR1*m clones 3 to 6 months preprogression [2]. The phase III PADA-1 study demonstrated that switching to fulvestrant based on ctDNA-detected *ESR1*m (without radiological progression) substantially extended median progression-free survival (PFS) versus continued AI (11.9 months vs. 5.7 months; hazard ratio [HR] = 0.61, 95% confidence interval [CI] = 0.43 to 0.86, *P* = 0.004) [3]. Similarly, the SERENA-2 trial demonstrated superior PFS with the novel oral selective estrogen receptor degrader (SERD) camizestrant compared to fulvestrant in *ESR1*-mutated ABC [4].

Bidard et al. [5] recently reported the pivotal results of the SERENA-6 trial (based on the initial data cutoff published in June 2025 *The New England Journal of Medicine* (NEJM); later-reported data are not discussed here). This double-blinded phase III study enrolled patients with HR^+^HER2^−^ ABC who had received at least 6 months of first-line AI plus CDK4/6i. *ESR1*m was assessed every 2 to 3 months via ctDNA. A total of 315 patients with *ESR1*m and no radiological progression were randomized 1:1 to continue AI plus CDK4/6i or switch to camizestrant (75 mg/d) plus CDK4/6i. The primary endpoint was investigator-assessed PFS, and the secondary endpoints included overall survival (OS) and safety. Stratification factors included site of metastasis (visceral or nonvisceral), timing of *ESR1*m detection, duration of prior AI and CDK4/6i (<18 months vs. ≥18 months), and type of CDK4/6i.

Camizestrant plus CDK4/6i markedly prolonged median PFS compared to continuing AI plus CDK4/6i (16.0 months vs. 9.2 months; HR = 0.44, 95% CI = 0.31 to 0.60; *P* < 0.0001). The 12- and 24-month PFS rates were 60.7% vs. 33.4% and 29.7% vs. 5.4%, respectively. Benefits were consistent across subgroups, including those with nonvisceral metastases (HR = 0.38, 95% CI = 0.25 to 0.56) and Y537S mutations (HR = 0.29, 95% CI = 0.17 to 0.47). The time to deterioration in patient-reported global health status was notably longer in the camizestrant plus CDK4/6i group, with a median of 21.0 months compared to 6.4 months in the AI plus CDK4/6i group (HR = 0.54, 95% CI = 0.34 to 0.84).

Safety analysis showed a higher rate of grade ≥3 adverse events in the camizestrant plus CDK4/6i group compared to that in the AI plus CDK4/6i group (60.0% vs. 45.8%), primarily driven by CDK4/6i-associated hematologic toxicities. The most common nonhematologic adverse event was photopsia, reported more frequently with camizestrant (20.0% vs. 7.7%). However, photopsia events were predominantly low-grade, with minimal functional impact and no associated structural ocular changes.

SERENA-6 validates ctDNA-guided management of endocrine resistance, using ctDNA-detected *ESR1*m to enable early treatment escalation. This approach extended median PFS by 6.8 months compared with continuing AI therapy (16.0 months vs. 9.2 months) and was associated with a 47% reduction in risk of deterioration in patient-reported global health status. These findings suggest progress in the management of ABC, although real-world implementation may face practical challenges.

First, OS data remain immature. As approximately half of patients were still on first-line therapy at interim analysis, the proportion receiving second-line treatment is not yet final, making definitive conclusions on long-term benefit premature. Moreover, subsequent chemotherapy or antibody–drug conjugate use was higher in the camizestrant plus CDK4/6i group (46% vs. 35%), which seems to suggest that patients in the camizestrant plus CDK4/6i group had more severe disease progression and required more intense treatment regimens to control the condition. At the same time, chemotherapy and antibody–drug conjugates may cause more severe adverse reactions, and this imbalance may weaken the early PFS benefits. SERENA-6 may also be underpowered to demonstrate a significant OS benefit. The absence of crossover to camizestrant upon radiological progression in the AI plus CDK4/6i group further limited assessment of whether switching at molecular progression improves OS (quality of life aside) versus waiting for clinical progression. Although crossover is uncommon in registrational trials, permitting it could have clarified short-term benefits, particularly in light of the short PFS seen with single-agent SERD after progression in EMERALD [6].

The “molecular progression” concept of SERENA-6 raises questions about optimal intervention timing. This “early resistance intervention” model—switching upon ctDNA *ESR1*m detection—appears superior to postradiographic “salvage therapy” in prolonging tumor control and maintaining quality of life. For instance, the EMERALD trial reported only modest PFS benefit with elacestrant (median 3.8 months) and no significant OS benefit (HR = 0.59, 95% CI = 0.36 to 0.96) [6]. In contrast, the ongoing phase III SERENA-4 trial (NCT04711252) is evaluating the first-line use of camizestrant plus CDK4/6i in treatment-naïve HR^+^HER2^−^ ABC [7]. Positive results could support broader camizestrant use, although this one-size-fits-all strategy risks overtreating those who never develop *ESR1*m.

An obstacle to ctDNA-guided management is the absence of harmonized analytical standards. Laboratories employ disparate technologies (digital polymerase chain reaction [PCR] vs. next-generation sequencing) and technical specifications, yielding divergent limits of detection [8]. Both the PADA-1 (in-house droplet digital PCR) and SERENA-6 (Guardant360 CDx [Guardant Health]) strategies operate at a limit of detection of around 0.2% to 0.5% variant allele frequency. However, interlaboratory concordance remains suboptimal due to differing technologies and protocols, complicating uniform result interpretation across centers. Less sensitive assays may miss early *ESR1*m emergence. Cost represents an additional obstacle: serial ctDNA testing is more expensive than conventional imaging, and limited reimbursement may restrict access [9]. Future efforts should focus on cross-platform validation and individualized monitoring based on mutation kinetics to reduce economic burden.

Long-term camizestrant use requires further study. Safety data from SERENA-2 and SERENA-6 indicated mostly grade 1 to 2 hematologic toxicity and low-grade photopsia. However, cumulative gastrointestinal toxicity with prolonged dosing remains unknown. Real-world evidence is needed to assess long-term (≥2 years) outcomes [10]. Optimal treatment duration must be defined, with potential discontinuation criteria including sustained undetectable *ESR1*m or prolonged imaging response, although these require prospective validation.

Overall, SERENA-6 provides compelling evidence that a ctDNA-guided switch to camizestrant is a promising strategy for *ESR1*-mutated HR^+^HER2^−^ ABC. Despite immature OS data and potential real-world challenges, this adaptive first-line strategy may prolong treatment efficacy without compromising tolerability.

## References

[1] Herzog SK, Fuqua SAW. *ESR1* mutations and therapeutic resistance in metastatic breast cancer: Progress and remaining challenges. Br J Cancer. 2022;126(2):174–186. 34621045 10.1038/s41416-021-01564-x PMC8770568

[2] Fribbens C, Garcia Murillas I, Beaney M, Hrebien S, O’Leary B, Kilburn L, Howarth K, Epstein M, Green E, Rosenfeld N, et al. Tracking evolution of aromatase inhibitor resistance with circulating tumour DNA analysis in metastatic breast cancer. Ann Oncol. 2018;29(1):145–153. 29045530 10.1093/annonc/mdx483 PMC6264798

[3] Bidard FC, Hardy-Bessard AC, Dalenc F, Bachelot T, Pierga JY, de la Motte RT, Sabatier R, Dubot C, Frenel J-S, Ferrero JM, et al. Switch to fulvestrant and palbociclib versus no switch in advanced breast cancer with rising *ESR1* mutation during aromatase inhibitor and palbociclib therapy (PADA-1): A randomised, open-label, multicentre, phase 3 trial. Lancet Oncol. 2022;23(11):1367–1377. 36183733 10.1016/S1470-2045(22)00555-1

[4] Oliveira M, Pominchuk D, Nowecki Z, Hamilton E, Kulyaba Y, Andabekov T, Hotko Y, Melkadze T, Nemsadze G, Neven P, et al. Camizestrant, a next-generation oral SERD, versus fulvestrant in post-menopausal women with oestrogen receptor-positive, HER2-negative advanced breast cancer (SERENA-2): A multi-dose, open-label, randomised, phase 2 trial. Lancet Oncol. 2024;25(11):1424–1439. 39481395 10.1016/S1470-2045(24)00387-5

[5] Bidard FC, Mayer EL, Park YH, Janni W, Ma C, Cristofanilli M, Bianchini G, Kalinsky K, Iwata H, Chia S, et al. First-line camizestrant for emerging *ESR1*-mutated advanced breast cancer. N Engl J Med. 2025;393(7):569–580. 40454637 10.1056/NEJMoa2502929

[6] Bidard FC, Kaklamani VG, Neven P, Streich G, Montero AJ, Forget F, Mouret-Reynier MA, Sohn JH, Taylor D, Harnden KK, et al. Elacestrant (oral selective estrogen receptor degrader) versus standard endocrine therapy for estrogen receptor-positive, human epidermal growth factor receptor 2-negative advanced breast cancer: Results from the randomized phase III EMERALD trial. J Clin Oncol. 2022;40(28):3246–3256. 35584336 10.1200/JCO.22.00338 PMC9553388

[7] Im S-A, Hamilton EP, Llombart Cussac A, Baird RD, Ettl J, Goetz MP, Iwata H, Joy AA, Neven P, Haddad V, et al. SERENA-4: A phase 3 comparison of AZD9833 (camizestrant) plus palbociclib, versus anastrozole plus palbociclib, for patients with ER-positive, HER2-negative advanced breast cancer who have not previously received systemic treatment for advanced disease. J Clin Oncol. 2021;39(15_suppl):TPS1101.

[8] Ignatiadis M, Sledge GW, Jeffrey SS. Liquid biopsy enters the clinic—Implementation issues and future challenges. Nat Rev Clin Oncol. 2021;18(5):297–312. 33473219 10.1038/s41571-020-00457-x

[9] Siravegna G, Marsoni S, Siena S, Bardelli A. Integrating liquid biopsies into the management of cancer. Nat Rev Clin Oncol. 2017;14(9):531–548. 28252003 10.1038/nrclinonc.2017.14

[10] Layman RM, Liu X, Li B, McRoy L, Brufsky A. Real-world palbociclib dose modifications and clinical outcomes in patients with HR+/HER2− metastatic breast cancer: A Flatiron Health database analysis. Breast. 2025;81: Article 104448. 40138984 10.1016/j.breast.2025.104448 PMC11986547

